# Chitotriose Enhanced Antitumor Activity of Doxorubicin through *Egr1* Upregulation in MDA-MB-231 Cells

**DOI:** 10.3390/md22010026

**Published:** 2023-12-29

**Authors:** Heng Li, Ke Ji, Peng Liu, Yan Geng, Jinsong Gong, Chao Zhang, Zhenzhong Ding, Zhenghong Xu, Jinsong Shi

**Affiliations:** 1 Key Laboratory of Carbohydrate Chemistry and Biotechnology, Ministry of Education, School of Life Sciences and Health Engineering, Jiangnan University, Wuxi 214122, China; liheng@jiangnan.edu.cn (H.L.); 6171504003@jiangnan.edu.cn (K.J.); gengyan@jiangnan.edu.cn (Y.G.); jinsonggong.bio@hotmail.com (J.G.); 2 Institute of Edible Fungi, Shanghai Academy of Agricultural Sciences, Shanghai 201403, China; liupeng_jnu@163.com; 3 Yangzhou Rixing Bio-Tech Co., Ltd., Gaoyou 225601, China; 13852548098@163.com (C.Z.); yzdxgxy@126.com (Z.D.); 4 National Engineering Laboratory for Cereal Fermentation Technology, Jiangnan University, Wuxi 214122, China; zhenghxu@jiangnan.edu.cn

**Keywords:** chitotriose, triple-negative breast cancer (TNBC), antitumor activity, RNA sequencing, early growth response 1 (*Egr1*)

## Abstract

Dietary supplementation is proposed as a strategy to reduce the side effects of conventional chemotherapy for triple-negative breast cancer (TNBC). Chitosan oligosaccharides (COS), a functional carbohydrate, have been identified to potentially inhibit cancer cell proliferation. However, a detailed investigation is required to fully understand its exact influence, particularly in terms of COS composition. The antitumor activities of COS oligomers and its monomer of glucosamine, when combined with doxorubicin separately, were evaluated in MDA-MB-231 cells. Chitotriose was identified to have the most significant synergistic effect. Preincubation with chitotriose was observed to promote the entry of doxorubicin into the cell nuclei and induce morphological changes in the cells. Mechanism analysis at the transcriptional level revealed that the early growth response 1 (*Egr1*) gene was a key regulator in enhancing the suppressive effect. This gene was found to modulate the activity of its downstream gene, growth arrest, and DNA damage-inducible alpha (*Gadd45a*). The role of *Egr1* was confirmed through a small interfering RNA test and function assay. These findings provide insight into the effect and underlying mechanism of chitotriose supplementation for TNBC therapy.

## 1. Introduction

Since 2020, breast cancer has been identified as the most frequently diagnosed cancer type among women. According to a 2020 report by the International Agency for Research on Cancer (IARC), over 2.26 million new cases of breast cancer and nearly 685,000 breast cancer-related deaths were recorded worldwide [1]. Triple-negative breast cancer (TNBC), constituting approximately 15% of all breast cancers, is a heterogeneous subtype characterized by high histological malignancy, significant metastatic potential, and a poor prognosis for overall survival [2,3]. The absence of estrogen receptors, progesterone receptors, and human epidermal growth factor receptor 2 (Her2) in the tumor makes it challenging to treat with targeted or endocrine therapies [4]. As a result, cytotoxic chemotherapy has remained the primary treatment for TNBC [5]. FDA-approved chemotherapy regimens such as anthracyclines, taxanes, and anti-metabolites, known for inducing an apoptotic cascade via altering DNA replication processes and damaging mitochondrial membranes, are recommended [6]. However, the adverse effects of chemotherapy are significant. Chemotherapy with anthracyclines and Her2-targeted drugs has been associated with cardiomyopathy and congestive heart failure, while taxanes often result in long-term neuropathy [7,8]. To mitigate such harmness, dietary supplements, including vitamins, minerals, phytochemicals, hormones, and herbs, are adopted by 45~80% of breast cancer patients [9,10].

Recent studies have sparked debates on the usage of dietary supplements. Some antioxidants were found to potentially interfere with the cytotoxic effect of antineoplastic agents on tumor cells by generating reactive oxygen species (ROS) [11]. An observational study related to a high-risk breast cancer clinical trial revealed a 41% increase in recurrence hazard with antioxidant use (vitamins C, A, and E; carotenoids; coenzyme Q10), which was also associated with a similar but weaker correlation with mortality [12]. Contrarily, a recent study on functional carbohydrates in cell lines (U2OS, Saos-2, KP-4) revealed that mannose administration potentiated cell apoptosis by downregulating MCL-1 and BCL-XL protein levels, thereby increasing the cells’ vulnerability to chemotherapy with cisplatin or doxorubicin [13]. Consequently, there remains uncertainty regarding the effects of dietary supplements. Factors including the source, constituent, and amount need to be further investigated. 

Prompted by current studies, questions have arisen regarding the influences of other natural carbohydrate resources on conventional chemotherapy. Chitosan oligosaccharides (COS), common oligosaccharides degraded from chitosan with a degree of polymerization (DP) ranging from 2 to 10 [14], have been confirmed as safe [15] and effective dietary supplements. A substantial body of evidence attests to their anticancer, anti-inflammatory, and significantly high antioxidant properties in experimental models [16,17]. Various mechanisms have been proposed, including enhancing immune stimulation action via increasing T cells and macrophage infiltration [18], inhibiting human renal carcinoma growth through ROS-dependent endoplasmic reticulum (ER) stress pathways [19], and suppressing colorectal cancer by activating the AMPK signaling pathway [20]. Given the biological compatibility and effectiveness of COS, it is worth investigating whether synergistic effects exist when COS is used as a dietary supplement for chemotherapeutic agents and how it may function therein.

In this study, the antitumor activities of COS oligomers (DP 2-7) and the monomer of glucosamine were evaluated separately and in combination with doxorubicin in MDA-MB-231 cells. Chitotriose, which demonstrated the most significant effect, was selected for further study. To elucidate this underlying molecular mechanism, both morphological features and gene expression were investigated. Gene mapping was conducted to explore genes correlating with changes in morphology and activity-regulated via the chitotriose addition. Target genes were then validated at both the transcription and translation levels. These results could contribute to a deeper understanding of the influence of COS supplementation against TNBC.

## 2. Results

### 2.1. Chitotriose Impaired the Growth of MDA-MB-231 Cells via Momentary Preincubation

An initial investigation was conducted on the antiproliferative effects of COS monomers and oligomers (DP 2-7) against MDA-MB-231 cells. Following the determination of the IC_50_ value of doxorubicin against MDA-MB-231 cells (Figure S1), COS and glucosamine (0~1000 μM) were employed for combined treatment. A significant inhibition of tumor cell proliferation was observed with COS and glucosamine, albeit with varying degrees (*p*-value < 0.001). Notably, combined groups employing chitotriose and chitopentaose separately with doxorubicin demonstrated superior inhibitory effects in a dosage-dependent manner, with the chitotriose group exhibiting a 13.6% higher inhibition than others (Figure S2). Chitotriose was subsequently selected for further detailed studies.

**Figure 1:**
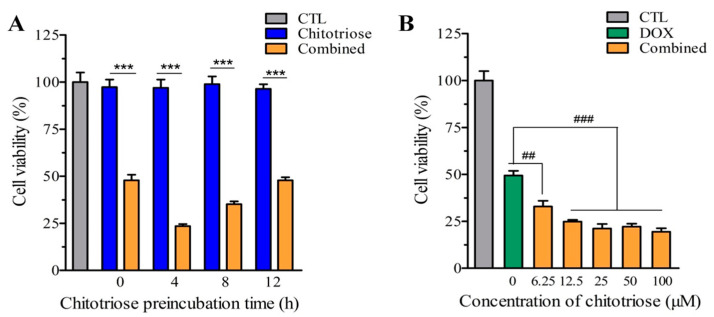
Effects of chitotriose on cell viability in MDA-MB-231 cells. (**A**) Preincubation time of chitotriose in combination with doxorubicin (doxorubicin 4.3 mM, treated for 24 h); (**B**) Concentration of chitotriose in combination with doxorubicin (chitotriose preincubation for 4 h, doxorubicin 4.3 mM treated for 24 h). Asterisks (***) indicate significant differences at a *p*-value < 0.001 between the combined and chitotriose groups. Sharps (^##^) and (^###^) indicate significant differences at *p*-values < 0.05 and 0.01 between the combined and DOX groups. There are 3 duplicates in each group.

The preincubation time and dose of chitotriose were examined. As depicted in Figure 1A, no cytotoxicity was observed when chitotriose was administered alone at the same concentration (100 μM). However, the significant inhibition of cell growth within 12 h was noted with combined treatment (*p*-value < 0.001). Interestingly, a higher inhibition ratio was achieved with shorter preincubation times. Cell viability for the combined treatment of chitotriose preincubated for 4 h was recorded at as low as 23.94%. No significant inhibition was observed when cells were administered simultaneously, suggesting a possible induction effect for momentary preincubation with chitotriose. Furthermore, preincubation with chitotriose at low concentrations (6.25 to 100 μM) enhanced the efficacy of doxorubicin in a dose-dependent manner. Even at a low concentration of 6.25 μM chitotriose, an enhancement in the inhibition ratio by 16.49% was observed compared with the group treated with doxorubicin (DOX group) (*p*-value < 0.001). The estimated lowest cell viability was 19.44% ± 1.91 with 100 μM of chitotriose. These data demonstrate an extraordinary synergistic effect with the combined use of chitotriose and doxorubicin in the combined group. 

### 2.2. Chitotriose Initiated Morphological Changes of MDA-MB-231 Cells

**Figure 2:**
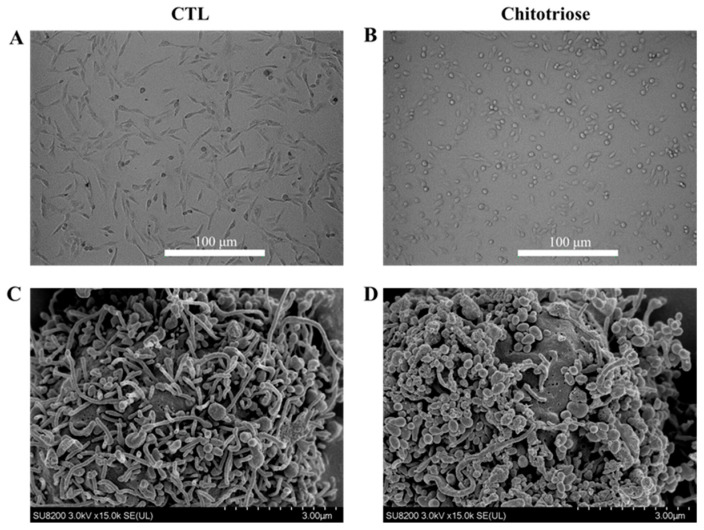
Morphological changes in MDA-MB-231 cells detected by a microscope and FESEM (*n* = 3 in each group). Experiments were repeated three times. (**A**) Microscope image of the CTL group; (**B**) Microscope image of the chitotriose group; (**C**) FESEM image of the CTL group; (**D**) FESEM image of the chitotriose group.

The effects of chitotriose preincubation on MDA-MB-231 cells were investigated using an optical microscope and field emission scanning electron microscopy (FESEM). A shift to a globular/spherical shape was observed in the cells after a 4 h chitotriose treatment (Figure 2B), signifying visible mesenchymal–epithelial transition (MET) behavior. This was markedly different from the elongated/spindle-like morphology of untreated MDA-MB-231 cells (Figure 2A). Further insights gathered from FESEM images showed a reduction in the length of the microvilli in cells treated with chitotriose. The CTL group, which displayed slender microvilli on the surface of MDA-MB-231 cells (Figure 2C), underwent a transition to shorter clusters following brief exposure to chitotriose (Figure 2D).

### 2.3. Chitotriose Enhanced Cell Apoptosis through Promoting Cellular Uptake of Doxorubicin

**Figure 3:**
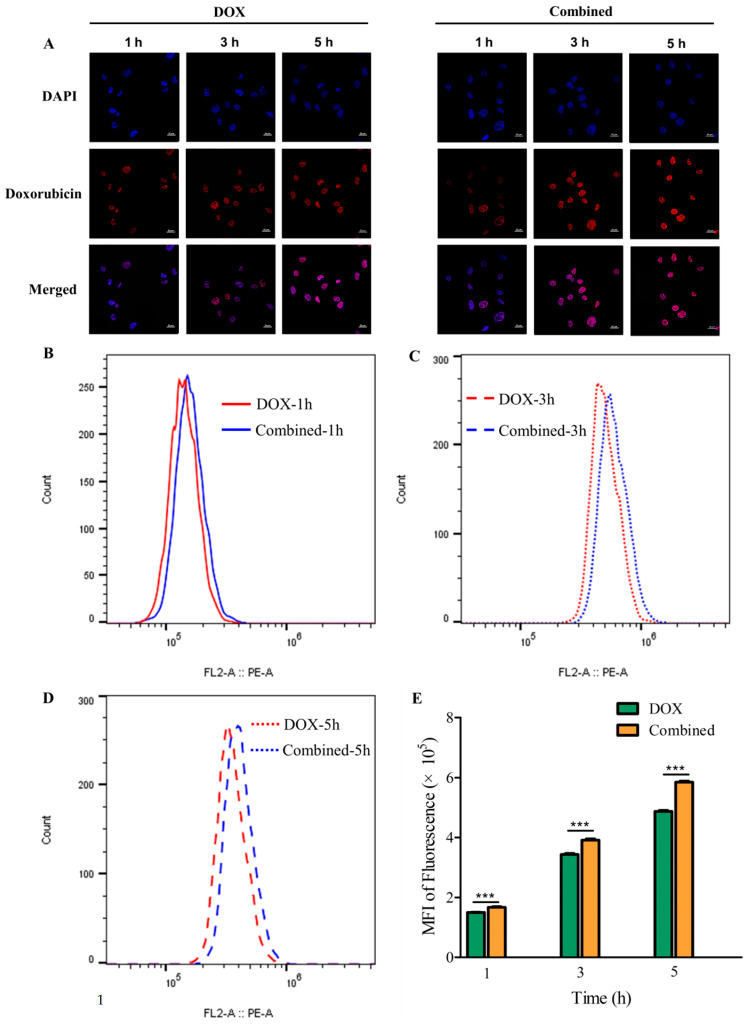
Cellular uptake assays performed using CLSM and flow cytometry on MDA-MB-231 cells (chitotriose preincubation for 4 h, doxorubicin at 4.3 mM treated for 24 h). Cellular uptake of doxorubicin with chitotriose preincubation depicted using CLSM (scale bars: 20 μm) (**A**); Intracellular fluorescence intensity of doxorubicin (**B**–**D**) detected via flow cytometry (n = 3 in each group); (**E**) Comparison of MFI. Asterisks (***) indicate significant differences at *p*-values < 0.001 between the combined and DOX groups.

The cellular uptake of doxorubicin in MDA-MB-231 cells following preincubation with chitotriose was assessed using confocal laser scanning microscopy (CLSM). As depicted in Figure 3A, minimal red fluorescence, indicative of cellular doxorubicin uptake, was observed after 1 h of preincubation with chitotriose. The location of the nucleus was marked by blue fluorescence via the 4,6-diamino-2-phenyl indole (DAPI) channel. A stronger red fluorescence signal was seen at the 3 h mark, suggesting a rapid increase in cellular doxorubicin uptake. Notable differences in fluorescence were still discernible between the DOX group and the combined group after 5 h.

Flow cytometry was utilized to provide quantitative data on the cellular uptake of doxorubicin (Figure 3B), and the ratio of mean fluorescence intensity (MFI) is displayed in Figure 3C. The results indicate that the MFI in both the DOX group and the combined group increased in a time-dependent manner at different detection times (1, 3, 5 h). Notably, the combined group showed a significant increase in MFI (*p*-value < 0.001), from 1.16-fold at 3 h to 1.17-fold at 5 h. This suggests that preincubation with chitotriose facilitated the cellular uptake of doxorubicin, thereby enhancing its inhibitory effect.

### 2.4. Tmem61 and Fgl2 Were Identified as Key Genes Correlated with MET

**Figure 4:**
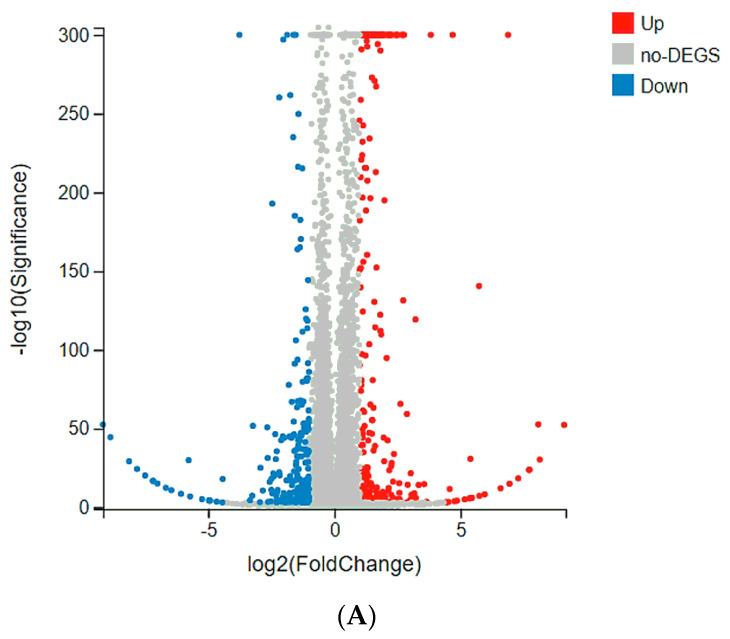

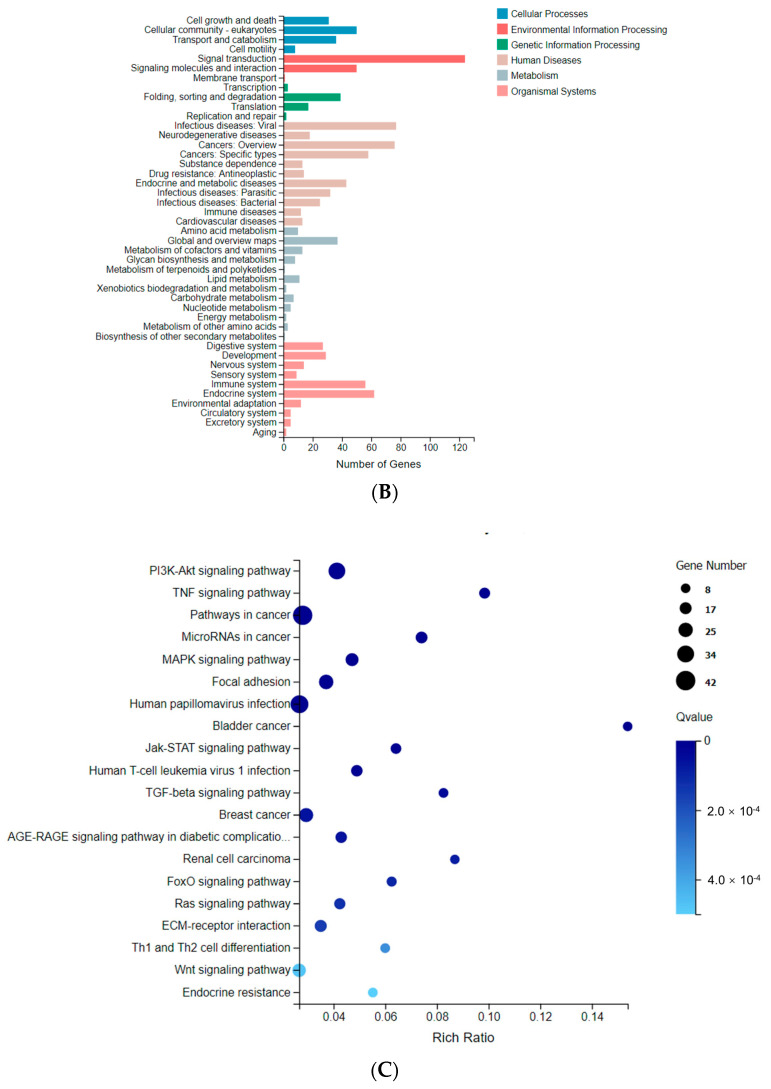
Target genes identified from chitotriose preincubation regulation (chitotriose preincubation for 4 h, doxorubicin 4.3 mM treated for 24 h, *n* = 3 in each group). (**A**) Volcano plot for the chitotriose group versus the CTL group. DEGs in each condition were identified (in red or blue), which was statistically significant. (**B**) KEGG pathway classification of all DEGs from Figure 4A. (**C**) KEGG pathway enrichment of the signal transduction pathway from Figure 4B.

To explore the underlying mechanism, a transcriptome analysis was performed. This aimed to identify target genes responding to chitotriose preincubation by analyzing differentially expressed genes (DEGs) between the chitotriose-treated group and the control group. As represented in Figure 4A, the volcano plot revealed a total of 554 DEGs, with 301 genes upregulated and 253 downregulated. A subsequent KEGG pathway classification was conducted for all identified DEGs (Figure 4B). The majority of these DEGs were associated with several categories, including signal transduction, viral infectious diseases, an overview of cancers, the endocrine system, and specific types of cancers. The signal transduction pathway, which contained the highest number of DEGs (124), underwent further annotation based on the q value (Figure 4C). The top five enriched pathways, listed in Table 1, were primarily involved in cancer pathways (42 DEGs), human papillomavirus infection (37 DEGs), the PI3K-Akt signaling pathway (33 DEGs), focal adhesion (25 DEGs), and breast cancer (24 DEGs). The top five DEGs in each pathway, determined via the absolute value of log2fold change (log_2_FC), were also presented. The unknown gene BGI_novel_G000649 emerged as the most significant DEG with a log_2_FC of −8.87. Among the annotated genes, *Tmem61* and *Fgl2* were identified as the most significant DEGs with log_2_FC values of 3.66 and −3.34, respectively. These genes were found to be enriched in the following three pathways: human papillomavirus infection, PI3K-Akt signaling pathway, and focal adhesion. Both *Tmem61* and *Fgl2* were enriched in the focal adhesion pathway, which has been studied for its role in regulating cell migration and dynamics [21]. This could be associated with MET induced via chitotriose. Additionally, the representative gene of *Cdh1* for mesenchymal–epithelial transition (MET) performance, encoding E-cadherin, was also detected (Table S2). The remaining gene *Colq* in the focal adhesion pathway had a log_2_FC of −1.95. Furthermore, *Myc* was found to be enriched in the PI3K-Akt signaling pathway and breast cancer pathway, with a log_2_FC of 2.10.

**Figure 5:**
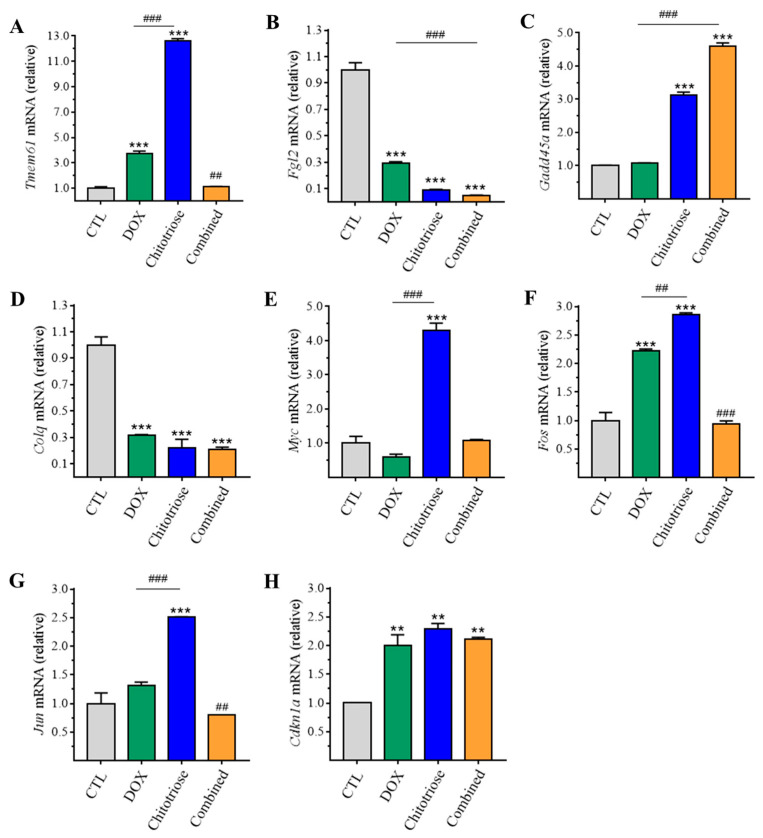
Validation of identified DEGs via RT-qPCR in MDA-MB-231 cells (n = 3 in each group). (**A**) *Tmem61*; (**B**) *Fgl2*; (**C**) *Gadd45a*; (**D**) *Colq*; (**E**) *Myc*; (**F**) *Fos*; (**G**) *Jun*; (**H**) *Cdkn1a*. Asterisks (**) and (***) indicate significant differences at *p*-values < 0.05 and 0.001 compared with the CTL group. Sharps (^##^) and (^###^) indicate significant differences at *p*-values < 0.05 and 0.01 between the combined and DOX groups compared with the DOX group.

**Figure 6:**
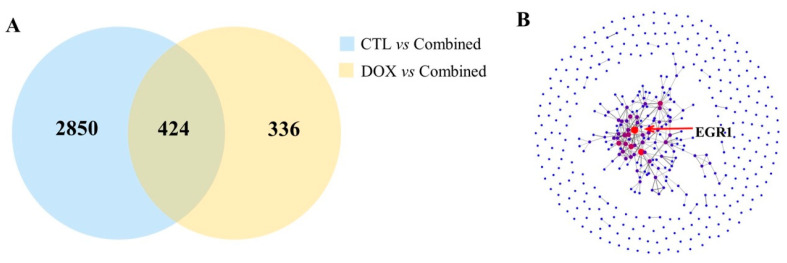
Target genes and the key protein identified from composite treatment (n = 3 in each group). (**A**) Venn diagram analysis indicated the number of genes of overlapping groups. In total, 411 common genes were further used for PPI analysis. (**B**) PPI analysis indicated EGR1 as the key regulatory protein.

To validate these DEG findings, RT-qPCR was performed. A correlation was constructed between the mRNA levels of DEGs from RNA-Seq and RT-qPCR data (Figure 5). In the chitotriose group, significant upregulation was observed in the mRNA levels of *Tmem61*, *Myc*, *Gadd45a*, *Fos*, *Jun*, and *Cdkn1a* (*p*-value < 0.001). On the other hand, *Fgl2* and *Colq* showed remarkable downregulation (*p*-value < 0.001). *Tmem61*, *Fgl2*, and *Colq* were found to be correlated with the focal adhesion pathway. Moreover, *Tmem61* and *Fgl2* exhibited the most prominent fold change between the chitotriose group and the CTL group at 12.62-fold and 11.36-fold, respectively (Figure 6A,B). However, no significant difference was noted in the mRNA levels of gene Tmem61 between the combined group and CTL group. In contrast, a different trend was observed for the DOX group (3.71 ± 0.23) and the chitotriose group (12.6 ± 0.16). Therefore, it can be inferred that the upregulation of *Tmem61* and downregulation of *Fgl2* might be involved in MET induced by chitotriose preincubation.

### 2.5. Upregulation of Egr1 Was Critical for Synergistic Effect of Composite Treatment

To investigate the underlying mechanism of chitotriose in enhancing the antitumor activity of doxorubicin, a correlation analysis was conducted between the chitotriose and combined groups. A Venn diagram was utilized to display the overlapping differentially expressed geness (DEGs) between the DOX group and the combined group, as well as between the CTL group and the combined group. Initially, 411 DEGs were screened (Figure 6A). Subsequently, a protein–protein interaction (PPI) network analysis was employed to predict protein interactions and identify the most extensively regulated genes. As depicted in Figure 6B, the proteins represented by the largest red dots displayed the most connections with others. EGR1 was identified as a key protein interacting with various other proteins, suggesting its potential role as a significant regulatory factor. Further analysis of the transcriptome data revealed that *Egr1* could also be identified with a high value of log_2_FC as 4.69 at the transcriptional level (Table S2). Therefore, the gene *Egr1* was selected for further investigation. In addition, the expression of the downstream gene *Gadd45a* was concurrently examined.

### 2.6. Downregulation of Egr1 with Small Interfering RNA (siRNA) Transfection Leading to Decrease in GADD45A

In order to further clarify the molecular mechanisms of *Egr1*, techniques such as RT-qPCR and Western blot were utilized. The transfection of MDA-MB-231 cells with si-EGR1 was performed to ascertain if *Egr1* was a pivotal gene involved in the regulation of the *Gadd45a* level.

**Figure 7:**
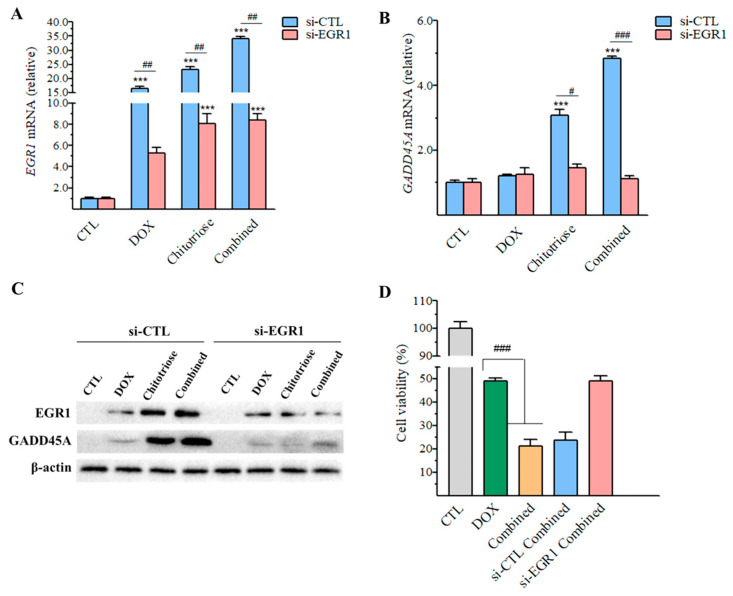
The effect of chitotriose on the transcription and expression levels of *Egr1* and *Gadd45a* (n = 3 in each group) with siRNA transfection. The mRNA level of (**A**) *Egr1* and (**B**) *Gadd45a* detected via RT-qPCR. Asterisks (***) indicate significant differences at *p*-value < 0.001 compared with the CTL group. Sharps (^#^), (^##^), and (^###^) indicate significant differences at *p*-values < 0.05, 0.01 and 0.001 for each group before and after siRNA transfection. (**C**) Protein levels detected using the Western blot assay. (**D**) Cell viability detected via the CCK-8 assay. Sharps (^###^) indicate significant differences at *p*-value < 0.001 between the combined and DOX groups.

As depicted in Figure 7A, a significant upregulation (*p*-value < 0.001) in the relative mRNA level of *Egr1* was observed across DOX, chitotriose, and the combined groups when compared with the CTL group. Following the interference of the gene expression via si-EGR1, a significant downregulation (*p*-value < 0.01) in the mRNA level of *Egr1* was noted, maintaining similar levels between the chitotriose group and the combined group. The efficacy of knock-down by siRNA could be inferred to be within the range of 65.41~75.40% based on the mRNA levels of *Egr1*. Concurrently, a decrease in EGR1 expression also signaled the success of si-EGR1 (Figure 7C).

Regarding the downstream gene *Gadd45a*, its mRNA level was significantly upregulated (*p* < 0.001) in the chitotriose and combined groups compared with the CTL group (Figure 7B). However, when EGR1 was targeted by si-RNA, the level of *Gadd45a* decreased by 2.10-fold in the chitotriose-treated group and 4.28-fold in the combined group, corroborating the synergistic effect with *Egr1*. The further detection of protein expression levels (Figure 7C) confirmed that targeting si-EGR1 evidently inhibited GADD45A expression in both the chitotriose group and the combined group while exerting a minimal effect on the DOX group.

To confirm whether chitotriose-induced EGR1 expression was involved in the synergistic inhibition of cell growth, a cell viability assay was conducted (Figure 7D). It was observed that both the normal combined group and si-CTL-combined group significantly inhibited cell growth (*p*-value < 0.001). The cell viability of the si-EGR1-combined group was recorded as 48.94% ± 2.32, indicating no significant difference when compared with the DOX group (49.10 ± 1.26). An examination of cell viability in composite treatment revealed that chitotriose played a crucial role in enhancing the apoptosis-inducing effect of doxorubicin. Overall, the results demonstrated that the downregulation of *Egr1*, which eliminated the synergistic effect, elucidated its key role in composite treatment with doxorubicin.

## 3. Discussion

Dietary supplements have been identified as a novel therapeutic strategy for Triple Negative Breast Cancer (TNBC), counteracting the excessive cytotoxicity associated with traditional chemotherapy. Chitooligosaccharides (COS) have demonstrated potential as functional carbohydrates with anti-tumor activity, capable of inducing G2/M apoptosis and S cell arrest in HCT116 cells at concentrations ranging from 0.3125 to 10 mg/mL [19]. Further studies have revealed that the IC_50_ of COS against various cancer cells, including HeLa, MCF-7, and H460 cells, are approximately 2.3, 2.0, and 4.1 mg/mL, respectively [22]. While COS has shown some degree of inhibitory function on tumor cells, the precise effects of each COS oligomer and their roles as dietary supplements remain to be elucidated.

In this study, COS oligomers and the monomer of glucosamine with defined structures were utilized. Despite the lack of apparent cytotoxicity against MDA-MB-231 cells, a notable enhancement in the inhibitory effect was observed when COS and glucosamine were combined with doxorubicin. This finding diverges from previously reported results, potentially attributable to the concentration of COS used. Previous studies reporting COS’s inhibitory effects on tumor cell proliferation commonly utilized high concentrations (typically at milligram levels), which could be excessively high when converted into human dosage. Notably, chitotriose demonstrated the most significant suppressive and dose-dependent effects on MDA-MB-231 cells when combined with doxorubicin, while others exhibited no clear trend. It could be seen that COS with different degrees of polymerization exerted different influences. In contrast, chitohexaose was reported to exert the greatest direct inhibitory activity among the five different COS oligomers (DP 2-6) at a concentration of 100 μg/mL on A549 cells treated for 48 h [23]. Therefore, the differential behavior of COS oligomers underscores the necessity of studying their structural properties [24], strong electric charge [25], molecular weight, and degree of deacetylation [26,27,28] across various tumor cells to gain a comprehensive understanding. It could be deduced that COS exerted complex effects on the cell, while the interaction between different signaling molecules might also have an impact. 

A combined treatment of COS (40, 80 mg/kg) and cyclophosphamide was administered to S180 residual tumor mice, demonstrating the potential of COS to enhance the antitumor effect in vivo [18]. Unfortunately, the development of scheme studies remains limited. Additionally, carbohydrates such as mannose exhibited a similar synergistic inhibitory effect with cisplatin or doxorubicin on cells at a low concentration of 25 mM [13]. This suggests that mannose might increase cell susceptibility by regulating the levels of anti-apoptotic proteins and phosphomannose isomerase. This indicates the potential induction effect of administered carbohydrates, which merits further in-depth exploration.

Another significant finding was the morphological changes associated with MET in cells preincubated with chitotriose. This change was directly linked to enhanced cell death when combined with doxorubicin. The addition of chitotriose amplified the suppressive effect by promoting the cellular uptake of doxorubicin. Doxorubicin can inhibit topoisomerase II, which plays a crucial role in DNA replication, recombination, and repair by penetrating the cell nucleus and integrating it into the DNA. Preincubation with chitotriose facilitated the rapid enrichment of doxorubicin in the nucleus, enhancing DNA intercalation, mitochondrial impairment, free radical formation, and oxidative damage [29,30]. This approach could potentially counteract the non-targeting property of doxorubicin to some extent. The synergistic effect of these two agents on the inhibition of MDA-MB-231 cells could be fully leveraged while minimizing side effects by reducing the dose of doxorubicin.

Leveraging the advantages of RNA sequencing in obtaining the sequence, structure, and expression of all cell or tissue transcripts and bridging genotyping and phenotyping [31], this study explored the molecular scheme of chitotriose preincubation and its subsequent synergistic efficacy when combined with doxorubicin. A comparison was first conducted between the chitotriose and CTL groups. Several DEGs were identified and further validated through RT-qPCR experiments, in which *Tmem61* (upregulated by 12.62-fold) and *Fgl2* (downregulated by 11.36-fold) were the most significant. *Tmem61* encodes transmembrane protein 61 (TMEM61), a protein with a largely unexplored function. However, related TMEM family members have been identified as either tumor suppressors or oncogenes. The expression of TMEM48 and TMEM97 are potential prognostic biomarkers for lung cancer, while TMEM45A and TMEM205 are implicated in tumor growth and invasion [32]. In this study, *Tmem61* was found to be upregulated at mRNA levels, suggesting its involvement in the synergistic inhibitory effect of tumor growth induced by chitotriose preincubation. The other gene, *Fgl2*, encodes fibrinogen-like protein 2 (FGL2), a transmembrane protein from the fibrinogen family [33] known for its substantial pro-angiogenic activity. The overexpression of FGL2 can induce epithelial–mesenchymal transition (EMT) and promote tumor progression [34,35]. In this study, *Fgl2* was downregulated after the cells were preincubated with chitotriose, suggesting that chitotriose may increase the cell’s susceptibility to damage by inducing differentiation into epithelial-like cells, a process that is in contrast to EMT. Therefore, FGL2 could potentially function as a regulator of cellular phenotype changes in MDA-MB-231 cell epithelial transformations. No other signs of apoptosis, such as nuclear fragmentation or apoptotic body formation, were observed, only a reduction in the microvilli length for MDA-MB-231 cells. Previous studies on COS’s impact on MDA-MB-231 cells reported that COS reduced matrix metalloproteinase-9 (MMP-9) secretion, thereby inhibiting tumor cell migration and invasion [36]. Further studies are required for the functional verification of these two genes.

To further elucidate the synergistic effect of chitotriose, PPI analysis was conducted between the combined and DOX groups. *Egr1* was identified as the key factor in response to this synergistic effect, demonstrating the most intense correlation with other proteins, which is also in accordance of transcriptome analysis result. The corresponding protein of early growth response protein 1 (EGR1), a well-known transcription factor, is capable of regulating signaling cascades, including differentiation, cell growth, and death [37,38]. *Egr1* is involved in p53 signaling, with *Gadd45a* as a downstream gene. EGR1 can initiate transcription and mediate the cell cycle, DNA repair, and apoptosis by directly binding onto the protein of growth arrest and the DNA damage-inducible protein alpha (GADD45A) promoter [39]. The overexpression of EGR1 has been revealed to act as an inhibitor in breast cancer [40]. Following momentary preincubation with chitotriose, both *Egr1* and *Gadd45a* expression levels in the combined group exhibited marked elevation. Interestingly, when *Egr1* was knocked down, a corresponding decline in the *Gadd45a* level was observed. This suggests that *Egr1* serves as a key upstream gene regulating *Gadd45a*, exhibiting a consistent trend at both transcriptional and expression levels.

Further validation through the cell viability assay confirmed that the upregulation of *Egr1* was instrumental in the synergistic anti-tumor activity observed in the composite treatment with doxorubicin. Consistent with our findings, the upregulation and nuclear translocation of *Egr1* has also been shown to induce cell death in prostate cancer cells [41]. Functional verification in our experiments indicated that *Egr1* could be a critical factor in the tumor suppressor pathway, capable of activating the downstream gene *Gadd45a* to induce cell apoptosis. Dialectically, *Egr1* also plays a dual role in facing different disease situations with different signaling pathways. It is reported that EGR1 can suppress transformation and counteract apoptosis through the coordinated activation of TGF-β1, FN, p21Waf1/Cip1, and FAK, which further lead to cell attachment enhancement and caspase activity reduction. Therefore, the exact effect of *Egr1* and its resulting influence needs further and detailed investigation [42].

## 4. Materials and Methods

### 4.1. Materials and Reagents

COS oligomers (DP 2-7) and glucosamine were purchased from Qingdao BZ Oligo Biotech Co., Ltd., (Qingdao, China), while the cell counting kit-8 (CCK-8) was procured from MedChemExpress, Inc. (Monmouth Junction, NJ, USA). The doxorubicin hydrochloride and Coomassie protein assay reagents were acquired from Sigma-Aldrich, Inc. (St. Louis, MO, USA). Dulbecco’s modification of Eagle’s medium (DMEM), the Trizol reagent, 4′,6-Diamidino-2-phenylindole dihydrochloride (DAPI), RNA-related kits, and the Lipofectamine RNAiMAX Transfection Reagent, as well as the radioimmunoprecipitation assay (RIPA) lysis buffer, were all obtained from Thermo Fisher Scientific Inc. (Waltham, MA, USA). The siRNA reagents were sourced from Santa Cruz Biotechnology (Santa Cruz, CA USA). The antibodies for early growth response protein 1 (EGR1) (catalogue number: 4154S), growth arrest, and the DNA damage-inducible protein alpha (GADD45A) (catalogue number: 4632S), and β-actin (catalogue number: 4970S) were procured from Cell Signaling Technology, Inc. (Danvers, MA, USA). All primers were synthesized by GENEWIZ Biotech Co., Ltd. (Suzhou, China).

### 4.2. Cell Culture

The human breast cancer cell line MDA-MB-231 was generously provided by Professor Jian Jin of Jiangnan University. The cells were cultured in DMEM supplemented with 10% fetal bovine serum (Gibco, Grand Island, NY, USA), streptomycin (1000 U/mL), and penicillin (1000 μg/mL, Gibco, Grand Island, NY, USA) in a fully humidified atmosphere at 37 °C with 5% CO_2_. The cells were subcultured every 3 days, with the passage number ranging from 15 to 31.

### 4.3. Grouping and Treatment Experiment Design

The medium-incubated group served as the control group (noted as CTL). The positive control group (noted as DOX) consisted of cells treated with doxorubicin at the concentration of the IC_50_ value. The dosage of doxorubicin (0.54~8.6 μM) was optimized first to determine IC_50_. The chitotriose group (noted as Chitotriose) referred to cells treated with chitotriose alone at doses of 6.25, 12.5, 25, 50, and 100 µM. The composite treatment groups (noted as Combined) referred to the combined use of chitotriose and doxorubicin with various doses at different times.

### 4.4. Cell Viability Assay 

Single-cell suspensions were prepared from MDA-MB-231 cells in the logarithmic growth phase. In total, 5 × 10^3^ cells were seeded into each well of a 96-well culture plate for the assay. COS oligomers with DP ranging from 2 to 7 and glucosamine at concentrations of 10, 100, and 1000 μM were evaluated and compared. First, cells were preincubated with different COS oligomers and glucosamine, respectively. Then, the cells were stimulated with 4.3 μM of doxorubicin (the IC_50_ value is referred to in Figure S1 in the Supporting Information file) for 24 h. The cell viability assay was performed in triplicate and examined using the CCK-8 method. Generally, 10 μL of the CCK-8 solution was added to each well for 1 h, and then the optical density value was read at a wavelength of 450 nm (OD_450_) to determine cell viability on a microplate reader (Multiskan, Thermo, USA). Cell viability was calculated using the following formula:

Cell viability (%) = “OD (experiment) − OD (blank)”/“OD (control) − OD (blank)” × 100%(1)

Chitotriose was selected based on the comparative experiment results. Further tests, including the time course (0, 4, 8, 12 h) and concentration effect (0, 6.25, 12.5, 25, 50, and 100 μM) for chitotriose preincubation, were investigated.

### 4.5. Field Emission Scanning Electron Microscopy (FESEM) 

MDA-MB-231 cells were initially incubated with or without 100 μM of chitotriose for a period of 4 h. The cells were then harvested and centrifuged at a speed of 750 rpm for a duration of 5 min. Following fixation in 2.5% glutaraldehyde and subsequent washing twice with the DPBS buffer, the cells underwent another round of fixation with 1% osmium tetroxide. Dehydration was accomplished with an ethanol gradient of varying concentrations (30%, 50%, 70%, 90%, and 100%). Micrographs were obtained utilizing an FESEM (HITACHI SU8220, Tokyo, Japan) with the critical point drying method employed.

### 4.6. Confocal Laser Scanning Microscopy (CLSM)

A total of 1 × 10^5^ MDA-MB-231 cells were seeded into a specialized confocal dish with a diameter of 10 mm. The cellular uptake of doxorubicin in both the DOX and combined groups was examined at intervals of 1, 3, and 5 h, respectively. The cells were fixed in 4% (*v*/*v*) paraformaldehyde for a duration of 15 min at room temperature. The nuclei were subsequently stained with DAPI (1 mg/mL). Fluorescent images were captured under a two-photon fluorescent microscope with excitation wavelengths of 360 nm and 488 nm (Leica, Wetzlar, Germany).

### 4.7. Flow Cytometry Analysis of Cellular Uptake 

A total of 2 × 10^5^ MDA-MB-231 cells were seeded into a 6-well culture plate. The cellular uptake of doxorubicin was examined at intervals of 1, 3, and 5 h in both the DOX and combined groups, respectively. Cells were collected via centrifugation at 750 rpm for a duration of 5 min and subsequently washed twice with Dulbecco’s phosphate-buffered saline (DPBS). Samples were analyzed using flow cytometry, with fluorescence detected at 561 nm using a FACScan (Beckman Coulter, Miami, FL, USA). Data were processed using FlowJo 9 software (BD Biosciences, San Jose, CA, USA).

### 4.8. RNA Isolation, Library Construction and Sequencing 

The total RNA was extracted using the Trizol reagent, with each group processed in triplicate. RNA-Seq and bioinformatics analysis were undertaken by the Beijing Genomics Institute (BGI), China. The mRNA library was assembled and sequenced on the BGISEQ-500 platform. Initially, low-quality raw sequencing data were filtered out, and clean reads were subsequently mapped to a reference genome using the HISAT/Bowtie2 tool, achieving a mean ratio of 94.60% (Genome Reference hg38_ucsc). Gene quantification was performed by calculating FPKM based on the expectation–maximization algorithm, known as RSEM [43,44]. The RNA sequencing (RNA-Seq) method was applied according to the Poisson distribution [45]. Genes that met the threshold of a *q* value < 0.05 and |log_2_FC| > 1 (FC > 2) were identified and screened as DEGs. 

### 4.9. RT-qPCR Analysis 

The concentration and purity of total RNA were assessed using NANODROP 2000 (Thermo Fisher Scientific, Inc., Waltham, MA, USA). cDNA was synthesized from the RNA sample employing M-MLV Reverse Transcriptase for RT-qPCR. The reaction program was set as follows: 25 °C for 10 min, 48 °C for 40 min, 95 °C for 5 min, and 12 °C indefinitely. RT-qPCR experiments were carried out in duplicates on 96-well plates using a PikoReal Real-Time PCR System and SYBR Select Master Mix for CFX (Waltham, MA, USA). The reaction program incorporated one cycle of 50 °C for 2 min and 95 °C for 10 min, followed by 40 cycles of 95 °C for 15 sec and 60 °C for 1 min. β-actin was used as an internal reference, and the relative mRNA levels were computed using the 2^−ΔΔCt^ method. The primers used are listed in Table S1.

### 4.10. siRNA Transfection

The siRNA targeting EGR1 was employed for the si-EGR1 group, while mock siRNA was utilized for the si-CTL group. MDA-MB-231 cells, grown to 60% confluence, were seeded into a 6-well culture plate. Subsequently, transfection was performed using Lipofectamine RNAiMAX for a duration of 48 h on the following day. The verification of the transcription and expression level of target genes in transfected cells was carried out through RT-qPCR and Western blot, respectively.

### 4.11. Western Blot Analysis 

MDA-MB-231 cells were cleansed with cold DPBS, followed by the addition of 200 μL of the RIPA lysis buffer. The cell lysates were subsequently centrifuged, and the protein concentration was gauged using the Coomassie protein assay reagent. Protein samples, each weighing 50 μg, were electrophoresed on 10–15% SDS-PAGE gels. The protein was then transferred onto nitrocellulose membranes, which were blocked in 5% skimmed milk in tris-buffered saline tween-20 (TBST) for 2 h at room temperature. Subsequently, these membranes were incubated with a primary antibody overnight at 4 °C. A horseradish peroxidase-conjugated secondary antibody was applied at 1:5000 dilutions for 1 h at room temperature, followed by three washes in TBST. β-actin served as the control for normalizing protein expression.

### 4.12. Statistical Analysis 

Experiments were performed in triplicate, and the results were presented as the mean ± standard error of the mean (S.E.M.). Significant values were compared to the controls using either Student’s *t*-test or one-way ANOVA, followed by a one-way ANOVA using the software Prism GraphPad 6 (GraphPad Software Inc., La Jolla, CA, USA). A value of *p* < 0.05 was considered significant.

## 5. Conclusions

The present study demonstrated that the supplementation of chitotriose could enhance the anti-tumor activity of doxorubicin. A brief preincubation with chitotriose expedited the accumulation of doxorubicin in the nuclei and induced MET morphological changes in MDA-MB-231 cells. RNA-Seq analysis for gene mapping highlighted two key genes, *Tmem61* and *Fgl2*, which correlated with MET, and *Egr1*, as a crucial upstream gene that contributed to the synergistic effect of the composite treatment. Ongoing studies are focusing on gene mining and analysis, including the annotation of unknown genes, to further elucidate the underlying mechanism.

## Figures and Tables

**Table 1:** DEGs enriched from the top 5 pathways in signal transduction (n = 3 in each group).

KEGG Term	Gene ID	log_2_ (Chitotriose/CTL)	Q (Chitotriose/CTL)
ko05200: Pathways in cancer	BGI_novel_G000649 (unknown)	−8.87	3.24 × 10^−45^
	*Frat1*	−2.39	2.31 × 10^−20^
	*Gadd45a*	1.66	6.16 × 10^−268^
	*Rasgrp1*	1.58	1.23 × 10^−4^
	*Bmp4*	−1.53	0
ko05165: Human papillomavirus infection	BGI_novel_G000649 (unknown)	−8.87	3.24 × 10^−45^
	*Tmem61*	3.66	5.99 × 10^−4^
	*Fgl2*	−3.34	4.24 × 10^−5^
	BGI_novel_G000366 (unknown)	2.88	6.41 × 10^−60^
	BGI_novel_G000947 (unknown)	2.34	1.34 × 10^−6^
ko04151: PI3K-Akt signaling pathway	*Tmem61*	3.66	5.99 × 10^−4^
	*Fgl2*	−3.34	4.24 × 10^−5^
	*Myc*	2.10	0
	*Efna4*	−1.80	2.03 × 10^−78^
	*Efna1*	−1.42	6.58 × 10^−33^
ko04510: Focal adhesion	*Tmem61*	3.66	5.99 × 10^−4^
	*Fgl2*	−3.34	4.24 × 10^−5^
	BGI_novel_G000984 (unknown)	2.16	6.04 × 10^−13^
	*Colq*	−1.95	1.66 × 10^−4^
	BGI_novel_G000897 (unknown)	1.68	4.08 × 10^−4^
Ko05224: Breast cancer	*Myc*	2.10	0
	*Gadd45a*	1.66	6.16 × 10^−268^
	*Fos*	1.51	5.78 × 10^−14^
	*Jun*	1.33	0
	*Cdkn1a*	1.19	0

## Data Availability

The data supporting the conclusions of this study are available upon request from the corresponding author.

## Supplementary Materials

The following supporting information can be downloaded at: https://www.mdpi.com/article/10.3390/md22010026/s1, Figure S1: Investigation of IC_50_ value of doxorubicin against MDA-MB-231 cells; Figure S2: Effects of COS monomer and oligomers (DP 2-7) combined with doxorubicin on cell viability in MDA-MB-231 cells; Figure S3: Original Western blot images; Table S1: Primers for RT-qPCR. Table S2: mRNA level of *Cdh1* and *Egr1* through RNA-Seq analysis.
