# Supplementary material for: Chitotriose Enhanced Antitumor Activity of Doxorubicin through Egr1 Upregulation in MDA-MB-231 Cells
Source: Mar Drugs. 2023 Dec 29;22(1):26. doi: 10.3390/md22010026 (PMC10821154; doi:10.3390/md22010026)

Table S1. Primers for RT-qPCR

Gene	Forward	Reverse
β-actin	CTCTTCCAGCCTTCCTTCCTG	CAGCACTGTGTTGGCGTACAG
Cdkn1a	TGTCCGTCAGAACCCATGC	AAAGTCGAAGTTCCATCGCTC
Colq	CTTCCTACGGGGAATCTGTGT	CAATGGCGTTTTGGGTGTTC
Egr1	GGTCAGTGGCCTAGTGAGC	GTGCCGCTGAGTAAATGGGA
Fgl2	AGATTGCTCTGACTACTACGCA	TGCCATGTTCTGGTGAAGTTG
Fos	GGGGCAAGGTGGAACAGTTAT	CCGCTTGGAGTGTATCAGTCA
Gadd45a	GAGAGCAGAAGACCGAAAGGA	CACAACACCACGTTATCGGG
Jun	TCCAAGTGCCGAAAAAGGAAG	CGAGTTCTGAGCTTTCAAGGT
Myc	GTCAAGAGGCGAACACACAAC	TTGGACGGACAGGATGTATGC
Tmem61	TGGTTGACATCCCCACTTACG	GGCATCCCTCGATCCACTT

Table S2. mRNA level of *Cdh1* and *Egr1* through RNA-Seq analysis

Gene ID	log_2(Chitotriose /CTL)	Q(Chitotriose /CTL)	Pathway Name
Cdh1	1.13	5.57E-05	ko05226//Gastric cancer; ko05216//Thyroid cancer; ko04371//Apelin signaling pathway; ko04514//Cell adhesion molecules(CAMs); ko05218//Melanoma; ko05219//Bladder cancer; ko04015//Rap1 signaling pathway; ko05200//Pathways in cancer; ko04390//Hippo signaling pathway; ko05100//Bacterial invasion of epithelial cells; ko05213//Endometrial cancer; ko04520//Adherens junction
Egr1	4.69	0	ko05166//Human T-cell leukemia virus 1 infection; ko04371//Apelin signaling pathway; ko04933//AGE-RAGE signaling pathway in diabetic complications; ko04928//Parathyroid hormone synthesis, secretion and action; ko04912//GnRH signaling pathway

Figure S1. Investigation of IC_{50} value of doxorubicin against MDA-MB-231 cell

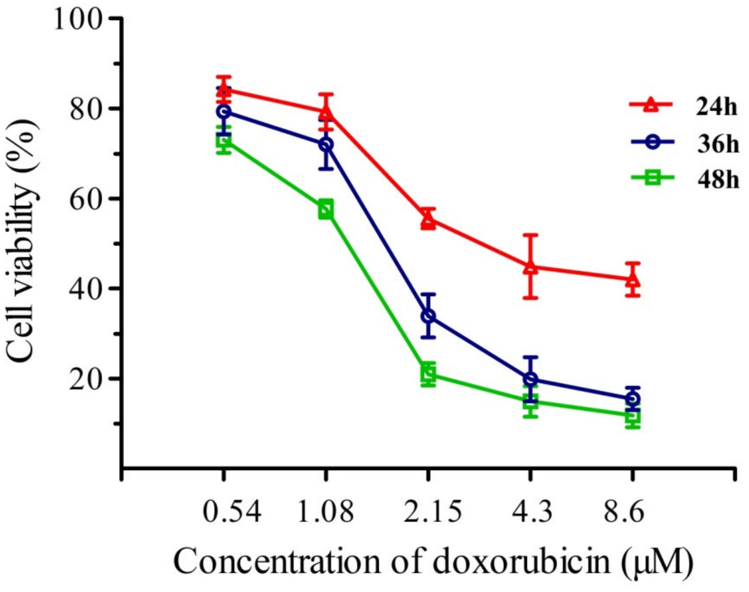

Figure S1

Figure S2. Effects of COS monomer and oligomers (DP 2-7) combined with doxorobicin on cell viability in MDA-MB-231 cells (n = 3 in each group). Asterisks (***) indicate significant differences at *p*-value < 0.001 compared with DOX group.

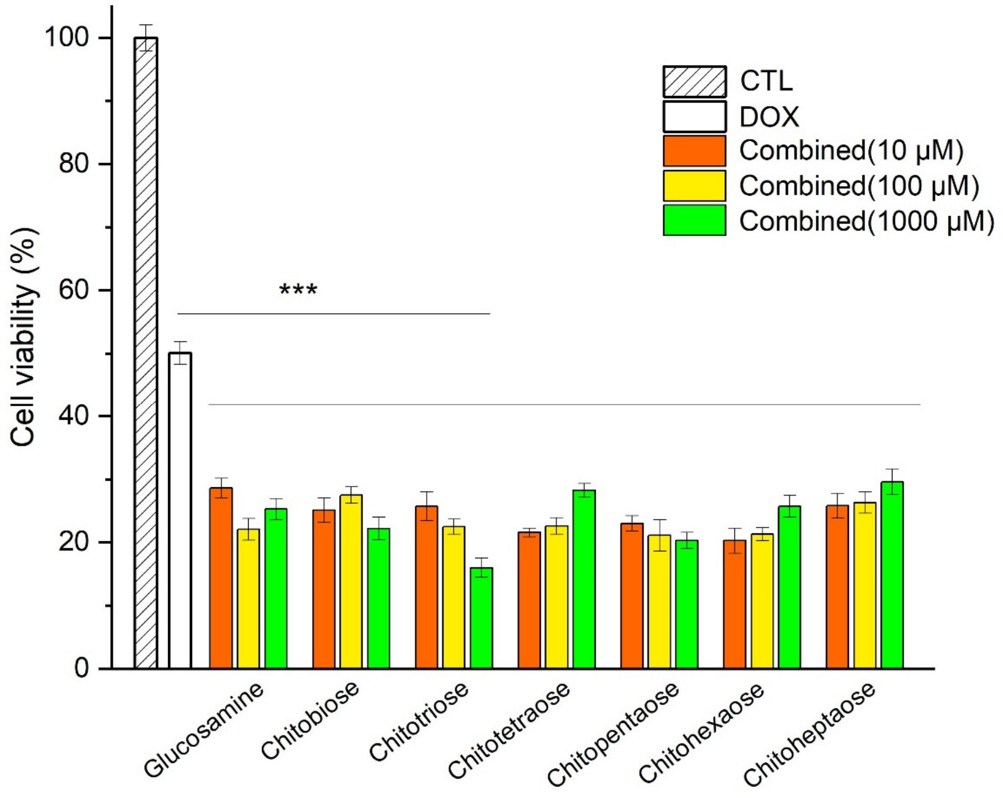

Figure S2

Figure S3. Original western blot images

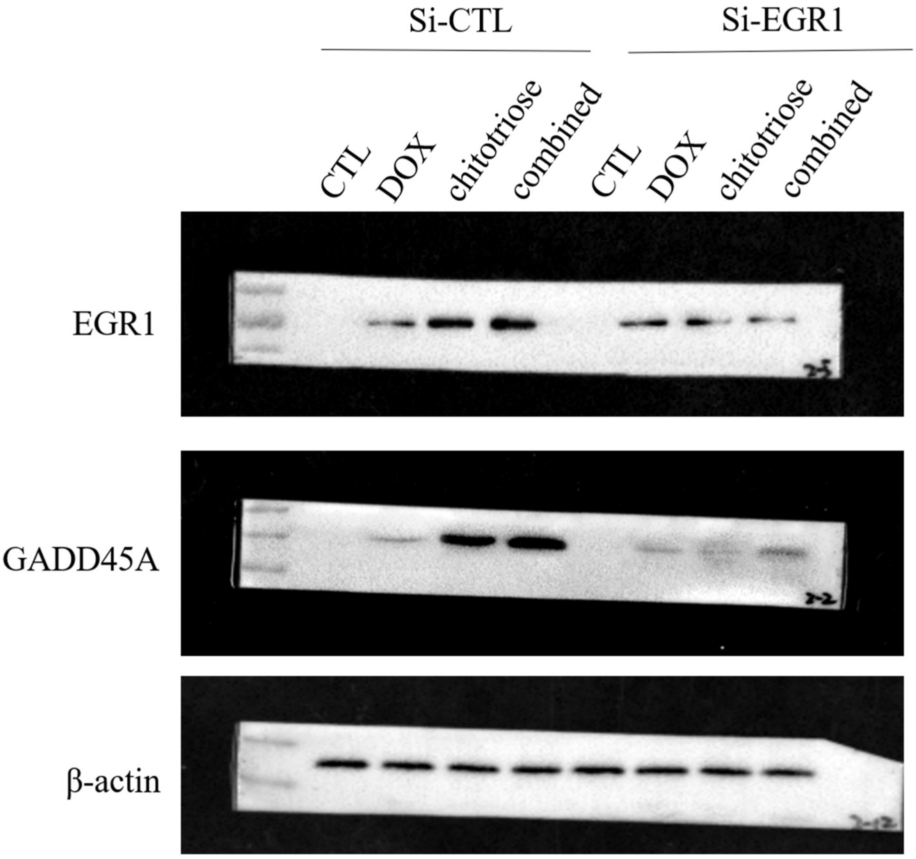

Supplement: Supplementary file 1 [file marinedrugs-22-00026-s001.zip › marinedrugs-2738253-supplementary.pdf]
